# Hearing aid effectiveness on patients with chronic tinnitus and associated hearing loss^☆^

**DOI:** 10.1016/j.bjorl.2022.03.002

**Published:** 2022-05-20

**Authors:** Patricia Simonetti, Laura Garcia Vasconcelos, Mara Rocha Gândara, Karina Lezirovitz, Ítalo Roberto Torres de Medeiros, Jeanne Oiticica

**Affiliations:** Universidade de São Paulo, Faculdade de Medicina, Hospital das Clínicas (HCFMUSP), Departamento de Otorrinolaringologia, São Paulo, SP, Brazil

**Keywords:** Hearing loss, Tinnitus, Hearing aids, Auditory rehabilitation

## Abstract

**Objective:**

Our study aimed to measure the effectiveness of using HA in reducing the disturbance caused by tinnitus.

**Methods:**

Study was designed as a within-subjects clinical trial. Nineteen patients with chronic tinnitus and untreated sensorineural hearing loss were under counseling, HA fitting and 6 months follow-up. Tinnitus assessment was performed with Tinnitus Handicap Inventory (THI), Visual Analog Scale (VAS), pitch and loudness matching, and Minimum Masking Level measurements (MML).

**Results:**

following 6 months of HA use, a reduction in reported tinnitus and hearing handicap scales scores was observed both statistically and clinically. The pitch and loudness matching, as well as MML at the baseline and final evaluation were compared. MML’s thresholds reduced significantly after 6 months of HA use.

**Conclusion:**

Our study has provided evidence that HA fitting is a valuable treatment strategy for chronic tinnitus relief and associated hearing loss subtype of patient.

**Level of evidence:**

3.

## Highlights

• Amplification restores auditory input and reduce tinnitus annoyance.
• THI reduction has been considered to be clinically and statistically significant after 6-months of hearing aid usage.
• A significant reduction in THI and VAS indicates a significant improvement in tinnitus severity.
• Minimal masking levels showed statistically significant reduction after 6-months use of HA, and inverse correlation with duration of tinnitus.
• THI, HHIA, VAS for tinnitus annoyance and MMLs are interrelated parameters on tinnitus amelioration.

## Introduction

Tinnitus is described as perception of a sound or noises in the ears or head, without a corresponding external acoustic stimulation. Tinnitus has a 10%‒17% world prevalence rate reported in various studies,^1^, ^2^ but this number can be upwards of 20%.^3^ Tinnitus is three times more prevalent among adults older than 65 years of age, affecting approximately 33% of those who also suffer from hearing loss.^4^, ^5^

Hearing loss is associated to tinnitus in more than 90% of the cases^6^, ^7^ and for that reason Hearing Aids (HA) have been usually provided as a first-choice treatment.^8^, ^9^ HA seem to be helpful for patients as they provide compensation for hearing loss, reducing listening and cognitive efforts in communication contexts. HA usage prevents sensory deprivation and may induce secondary plasticity in the Central Nervous System (CNS),^10^, ^11^, ^12^ Additionally, its use also significantly reduces tinnitus-related perception and annoyance, by promoting partial or total masking of it.^13^, ^14^

Although those benefits have been pointed out in recent reviews, there is still lack of strong evidence to recommend HA for tinnitus bothersome and magnitude mitigation.^15^, ^16^ Different outcomes tools, mostly self-reported, small samples, short term studies, lack of tinnitus objective measurement variables and the absence of control groups are among the reasons.^17^, ^18^

Our study aimed to measure the effectiveness of using HA in reducing the annoyance and disturbance caused by tinnitus, as well the perceived handicap due to hearing loss, and correlate those rates with tinnitus psychoacoustic measurements.

## Methods

The study was approved by the local medical ethics committee under protocol number 611.174 in April, 2014 and Clinical trials registry number: NCT03657615. The present study was designed as a within-subjects clinical trial. After the informed consent, nineteen subjects were included based on the following criteria: chronic perception of tinnitus for a duration of more than 6 months; tinnitus perception categorized by Tinnitus Handicap Inventory (THI, translated and validated to Brazilian Portuguese language)^19^ as moderate, severe or catastrophic (38 or greater score); mild and moderate degree of bilateral and symmetric hearing loss (defined as auditory thresholds more or equal 20 dB Hearing Level (HL) over frequencies (250–8000 Hz), and not exceeding 70 dB HL). Subjects had no previous experience with HA. Symmetrical hearing loss was defined as not more than 10 dB HL in two or more frequencies or more than 15 dB HL in one frequency, compared across both ears. Hearing loss configuration and severity were based on Silman and Silverman, 1997^20^ and Bureau International d’Audio Phonologie (BIAP) 1997 criteria.^21^

Individuals with unilateral, mixed, or conductive hearing loss, with pulsatile or myoclonic tinnitus were excluded. Participants were further screened for depression and anxiety using the Generalized Anxiety Disorder 7-item (GAD-7) and the Patient Health Questionnaire (PHQ-9). Subjects who scored higher than seven on GAD-7 or greater than nine on PHQ-9 were also excluded,^22^, ^23^ as they may be indicative of current states of depression or anxiety and would require additional pharmacological or other tinnitus treatment.

### Procedures

All the procedures described below were performed after medical evaluation and a complete tinnitus evaluation protocol, which included somatosensory assessment, self-related questionnaires for tinnitus, THI^24^, for hearing (Hearing Handicap Inventory for Adults – HHIA),^25^, ^26^ and for tinnitus annoyance (Visual Analogue Scale – VAS). THI, HHIA, and VAS were applied on baseline, and follow-up visits at 1^st^month, 3^rd^month and 6^th^month (final measure).

All subjects underwent pure tone audiometry (250–8000 Hz air and bone conduction testing frequencies), speech thresholds and recognition tests, immittanciometry and stapedian reflexes measurements. Audiometric earphones (TDH 39 series- Telephonics® were used, tested in a soundproof chamber using an Itera II audiometer (Madsen Aurical ICS, USA), calibrated under ANSI S 3.6-1996 normalization.

Tinnitus pitch and loudness matching, and Minimum Masking Levels tests (MML) were performed as baseline and final measurement (after 6 months of HA usage). The procedure followed the standardized method: presenting 2 stimuli so the patient could choose the one that most closely resembles his tinnitus. All of them were replicated three times and the mean value was determined as definitive. We used a tonal or narrow band noise stimulus to match tinnitus depending on the sound characteristics reported by the patient. After pitch and loudness being determined we searched for the minimal tone or noise in that frequency that patient could no longer listen to his tinnitus, and that was the final MML threshold computed.

All subjects underwent an individual counseling session and received a detailed explanation about the results of their clinical and audiological evaluation. They were provided with hearing physiology and tinnitus generation information; some habituation concepts were also explained. Counseling was provided whenever they needed during the following appointments. The same audiologist performed all the counseling to all subjects.

All patients were fitted bilaterally for hearing loss compensation. HA fitting, adjustments, real ear measurements and follow-up were performed by the same audiologist. Apart from different HA used, device selection followed strict criteria to fit hearing loss demands, patient profile, resources and algorithms recommended for tinnitus patients. Those characteristics are: (a) Digital Behind-the-ear HA with wide frequency response range; (b) Wide dynamic range compression (WDRC); (c) Maximum output limiting; (d) Feedback cancellation system to allow open ear fittings; (e) Data log to verify HA use.

Other algorithms and resources such as sound generators and noise suppressors were available but were not used during the study. To prescribe and verify acoustic response, we used the HA fitting protocol recommended by the National Acoustics Laboratories, NAL NL-2.^27^ The following devices were provided: Verso TS 762DRW (GN RESOUND®), Taurus 175D (Beltone®), MENU5-m e MENU10-m (WIDEX®), Orion 2S a Octiv+ (Sivantos®). After fitting, all patients had regular follow-ups, including medical and audiologist visits at 1st, 3rd, 6th month) to make fine tuning adjustments, check proper HA usage. HA fitting evaluation depended on each patient’s personal perception of benefit, speech recognition tests with and without HA, and real ear measurements.

### Statistical analysis

Categorical variables were described by their frequency of distribution. Quantitative variables (age, MML, THI, HHIA, VAS) were described by their mean, median and Standard Deviation (SD). Statistical associations for loudness, pitch and MML before and after HA use were performed by paired Wilcoxon test^28^ and for THI, HHIA and VAS by Friedman test^29^ assuming a 5% level of statistical significance (*p* < 0.05).

## Results

**Figure 1:**
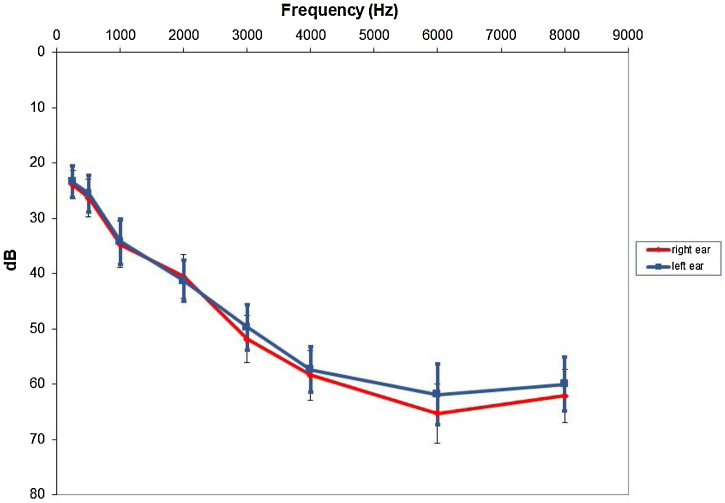
Hearing loss configuration for both ears (mean and standard error). dB, Decibels.

Our sample consisted of 10 females and 9 male subjects ranging from 32 to 62 years old (mean = 47.8, SD = 9.1). Most patients had bilateral chronic tinnitus (n = 17) with only two patients reporting unilateral tinnitus. Tinnitus duration varied from two to 40 years (mean = 12.3 and SD = 10.1). Patients presented with moderate (n = 11), severe (n = 4) or catastrophic (n = 4) chronic tinnitus according to THI classification. The VAS tinnitus annoyance scale had a mean of 8.4 (SD = 1.25). Pitch and loudness matching, as well as MML before HA fitted are shown in Table 1. Hearing loss configuration for all subjects are illustrated (Fig. 1) below.

**Table 1:** Tinnitus pitch, loudness and MML before HA use.

Variable	Mean	SD	Median	p25; p75
Tinnitus pitch (Hz)	6692	± 3584.6	6000	(4000; 8000)
Tinnitus loudness (dBSL)	6.1	± 4.5	5	(3; 8)
MML (dBSL)	6.4	± 3.2	6	(3; 9)

SD, Standard Deviation; p25, Percentile; p75, Percentile; dBSL, Sensation Level in Decibel; MML, Minimal Masking Level.

After the HA fitting session, patients were followed for counseling, hearing aid fine adjustments and check of proper HA usage. Time records or data log, which is an algorithm available on the HA selected for this study, showed a gradual increase in usage during the 6 months: 7.2 h per day (mean) ±3.1 in the 1st month and of 9.1 h per day (mean) ±3 in the 6th month.

**Figure 2:**
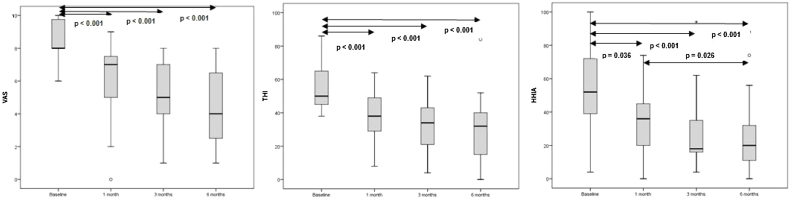
Behavioral results evaluation over time (baseline, 1st, 3rd, and 6th m HA use).

Results of self-assessment questionnaires THI, HHIA, their respective subscales and VAS were described according to the follow up evaluation moment: baseline, 1st month, 3rd month and 6th month (final measure), as seen on Table 2. Graph 1, 2 and 3 on Fig. 2 shows scores over time and reports on their statistical significance for multiple comparisons (*p* < 0.008). THI, HHIA, its subscales and VAS all showed a statistically significant score reduction (*p* < 0.001) along the 6 months follow up. Friedman’s tests were followed by multiple non-parametric comparisons for paired data. Thus, we were able to evaluate between what time points the differences occurred. As shown on Fig. 2, there was a statistically significant reduction of the initial results for the first month of HA usage for all questionnaires evaluated, (*p* < 0.001). The emotional and catastrophic domains of THI also showed greater reduction when we compared the initial results with the results of the third and sixth months, respectively. The HHIA social domain only showed statistical difference of the initial measurement in the third and sixth month of HA use.

**Table 2:** Behavioral results evaluation over time (baseline, 1^st^, 3^rd^, and 6^th^ m HA use).

	Baseline	1st m	3rd m	6th m	*p*
**VAS**					**<0.001**
mean ± SD	8.4 ± 1.2	6.2 ± 2.3	5.3 ± 2.1	4.5 ± 2.4	
median (p25; p75)	8 (8; 10)	7 (5; 8)	5 (4; 7)	4 (2; 7)	
**THI**					**<0.001**
mean ± SD	56.9 ± 15.5	37.6 ± 14.7	33.7 ± 16.3	30.5 ± 20.8	
median (p25; p75)	50 (44; 66)	38 (28; 50)	34 (20; 44)	32 (14; 40)	
**THI Functional**					**<0.001**
mean ± SD	26.7 ± 7.4	16.7 ± 7.0	15.2 ± 7.5	13.4 ± 9.2	
median (p25; p75)	24 (22; 32)	16 (12; 24)	14 (10; 20)	12 (4; 22)	
**THI Emotional**					**0.001**
mean ± SD	20.8 ± 7.1	13.7 ± 6.8	11.5 ± 8.0	11.0 ± 8.0	
median (p25; p75)	18 (16; 26)	14 (8; 16)	10 (6; 18)	12 (6; 16)	
**THI Catastrophic**					**0.006**
mean ± SD	10.2 ± 4.9	7.1 ± 4.3	7.4 ± 3.8	5.7 ± 5.8	
median (p25; p75)	10 (6; 14)	8 (4; 10)	8 (4; 10)	2 (0; 10)	
**HHIA**					**0.005**
mean ± SD	54.9 ± 27.0	33.4 ± 20.7	28.7 ± 22.3	26.7 ± 23.4	
median (p25; p75)	52 (36; 72)	36 (16; 46)	18 (16; 38)	20 (8; 34)	
**HHIA Social**					**0.004**
mean ± SD	26.8 ± 13.1	17.5 ± 11.8	14.6 ± 12.2	12.9 ± 10.7	
median (p25; p75)	24 (18; 36)	18 (4; 24)	10 (6; 24)	10 (6; 16)	
**HHIA Emotional**					**0.001**
mean ± SD	28.1 ± 14.6	15.8 ± 10.4	14.1 ± 11.5	13.2 ± 12.3	
median (p25; p75)	26 (16; 40)	16 (8; 22)	10 (8; 18)	10 (4; 16)	

m, Month; p, Statistically significant level; VAS, Visual Analogue Scale; SD, Standard Deviation; p25, Percentile; p75, Percentile; THI, Tinnitus Handicap Inventory; HHIA, Hearing Handicap Inventory for Adults; Friedman’s test.

The pitch and loudness matching, as well as MML at the baseline and final evaluation were compared and showed in Table 3. MML’s thresholds reduced significantly after 6 months of HA use (*p* = 0.047). However, tinnitus loudness, even though it decreased, did not show a statistically significant reduction. In fact, increases of no more than 2 dB NS were observed in 5 individuals. Interestingly, we also observed changes in the tinnitus pitch in nine individuals: lowering (n = 5) or increasing (n = 4) in the frequency, but there was no change for the other 10 patients.

**Table 3:** Pitch, Loudness and MML pre-X post 6 months of HA use.

	Baseline	Final	*p*
**Pitch (Hz)**			0.507^a^
mean ± SD	6692 ± 3584	6157 ± 3197	
median (p25; p75)	6000 (4000; 8000)	6000 (4000; 8000)	
**Loudness (dBSL)**			0.297^a^
mean ± SD	6.1 ± 4.5	5.0 ± 2.2	
median (p25; p75)	5 (3; 8)	4 (3; 7)	
**MML (dBSL)**			**0.047**^a^
mean ± DP	6.4 ± 3.2	5.2 ± 2.7	
median (p25; p75)	6 (3; 9)	5 (3; 7)	

*p*, statistically significant level; Hz, Hertz; SD, Standard Deviation; p25, Percentile; p75, Percentile; dBSL, Sensation Level in Decibel; MML, Minimal Masking Level.

a Wilcoxon paired test.

Our analysis revealed statistically significant direct correlation between reductions on THI total score and its emotional domain scale reduction and MML, HHIA (total) and its emotional domain scale (r > 0 and *p* < 0.05). THI functional domain scale reduction showed a statistically significant direct correlation with HHIA and its emotional domain, as seen in Table 4.

**Table 4:** THI reductions correlations.

		THI reduction	THI Functional reduction	THI Emotional reduction	THI Catastrophic reduction
**VAS reduction**	r	0.350	0.284	0.262	0.411
	p	0.142	0.239	0.279	0.080
**LOUDNESS reduction**	r	0.092	−0.004	0.237	0.005
	p	0.707	0.987	0.328	0.985
**MML reduction**	r	0.488	0.390	0.528	0.312
	p	**0.034**	0.099	**0.020**	0.194
**HHIA reduction**	r	0.457	0.485	0.504	0.034
	p	**0.049**	**0.035**	**0.028**	0.889
**HHIA Social reduction**	r	0.398	0.443	0.445	−0.068
	p	0.092	0.058	0.056	0.783
**HHIA Emotional reduction**	r	0.481	0.501	0.491	0.153
	p	**0.037**	**0.029**	**0.033**	0.533

THI, Tinnitus Handicap Inventory; VAS, Visual Analogue Scale; HHIA, Hearing Handicap Inventory for Adults; MML, Minimal Masking Level; Spearman’s correlation.

## Discussion

As hearing loss is presented in most tinnitus patients, HA seems to be always an eligible choice for rehabilitation. The idea is that by restoring auditory input, central nervous system gain, and neural hyperactivity may reduce, stimulate and build up secondary brain plasticity, as well as provide a permanent benefit on reducing tinnitus awareness.^30^ The limited evidence of HA effectiveness on tinnitus treatment in systematic reviews,^15^, ^16^ may possibly remain on methodological factors. Those include not standardized outcome measurement tools, non-specific or placebo effects, failure to control bias in large trials due to heterogeneity of symptoms and associated comorbidities affecting tinnitus patients, and, above all, the absence of controlled and randomized studies.^17^, ^18^ Although our clinical trial had been well designed to measure the effectiveness of HAs for tinnitus treatment, we have to point out some limitations of it. First, the absence of a control group with tinnitus and hearing loss, only undergoing counseling, for example, Second, the large heterogeneity of symptoms and comorbidities that are present in tinnitus patients required our selection criteria to be strict, resulting in a small but consistent sample. Controlling for the degree of hearing loss could have revealed whether the results would be applicable to patients with different degrees of hearing loss; And even though we used psychoacoustic measurements as an outcome measure, in addition to three other standardized tools, the absence of tinnitus measurements at a later follow-up (12 and 24 months) must be considered also as limitation.

Nevertheless, many authors had reported substantial benefits in HA fitting for tinnitus perception.^13^, ^31^, ^32^, ^33^ One of the major criticisms leans on the fact that the reduction of the hearing impairment by itself may justify improvements on communication skills, better quality of life referred by patients, as well as tinnitus distress alleviation and decrease on THI scores. Obviously, HA constant technology advances have made possible more effective fittings for both hearing loss restoration and tinnitus treatment. In our study, there are numerous factors that might have influenced the effectiveness of HA in reducing tinnitus perception. Among those we can mention the degree of high frequency hearing loss, the presence of mild or moderate hearing loss in the standard testing frequency, as well as the preservation of normal hearing thresholds in the low frequencies. McNeill and colleagues in their study have already made such a unique observation.^14^

HHIA scores on social and emotional domains showed a significant reduction over the 6 months of HA use, in agreement with patients continued and gradual clinical improvement. The social domain identifies everyday situations where speech comprehension is necessary for effective communication. Measures of impairment in the social domain reduced significantly when measured in the 6th month compared to the 1st month, in contrast to the emotional domain, which reduced significantly from baseline to the 1st month of HA use. We can point out some reasons for that including the acclimatization period which can take up to 2 months,^34^ and the HA fine tuning which improved progressively over the follow-up visits. So, the benefit provided by amplification takes longer to be fully experienced as it involves central nervous system plasticity, but the sense of care and initial relief provided by hearing restoration already appears in the first month of HA use, as seen on emotional domain score results.

Results of our study also shed light on the effective use of HA. Besides a validated HA prescription protocol and all the recommended fitting procedures, the increased HA usage seen in patient follow-up may explain the hearing skills improvement as well as the reduction of tinnitus distress.^35^ This point reinforces the importance of patient education and adherence. It's not uncommon they blame tinnitus other than hearing loss for their difficulties. Counseling, performed individually for all patients at the beginning of the intervention and at follow up visits, if necessary, was also crucial for HA acceptance and adoption.

The THI is the most used questionnaire in tinnitus clinical research worldwide, given its robustness, reproducibility,^36^ internal consistency and stability, and ease of application. Although it was initially created to classify the patient according to his reactions, its psychometric characteristics allow it to be used as a follow up instrument for evaluating tinnitus treatment results. A reduction of 20 or more points in the THI has been considered to be clinically significant.^37^ Such magnitude of reduction in total THI scores was observed in our study in 13 patients. In other words, there was a 2-level decrease in tinnitus grade severity, e.g., from catastrophic, severe, moderate to mild or slight symptom. Recently, a multicenter study (n = 200) has stated that a minimum 7-point reduction may already indicate some level of improvement and a reduction of 17-points or more would point out to highly significant improvement.^38^

A significant reduction in THI scores implies a significant improvement in tinnitus severity. The VAS is a metric scale usually used in most tinnitus clinical trials.^39^ In our study VAS was used to measure tinnitus annoyance, and it presented a significant score reduction over the 6 months follow up. Our results are in most part consistent with a randomized clinical trial^31^ that compared two sound therapy strategies in tinnitus patients: open digital HA versus a sound generator. The authors adopted the same variable tools (THI and VAS) and showed a highly significant improvement in both groups in the 3rd month of therapy. They reported a statistically and clinically significant tinnitus reduction measured by those variable scores, every 3 months, which continued up to 1 year of follow up.

Psychoacoustics measurements are performed as part of the tinnitus clinical evaluation for many reasons. Loudness and MML are valid measures of sound perception as they estimate tinnitus magnitude and can be used after intervention to evaluate their immediate effect, for example, after sound therapies strategies or magnetic transcranial stimulation techniques. Those measures are still recommended as a parameter for evaluating the results of tinnitus long-term interventions.^17^, ^40^ Changes in tinnitus pitch, on the other hand, may point out a sign of central nervous system tonotopy reorganization and neural plasticity.^41^ In our study after 6 months of HA usage almost half of our patients (47%) reported pitch shift.

In our study, tinnitus MML measurements showed statistically significant reduction, which may possibly indicate a permanent benefit of hearing amplification on tinnitus magnitude for some patients, once lower levels of sound are needed to mask tinnitus perception. Since duration of tinnitus varied among subjects, we also tested if it was correlated with any changes in measured variables such as THI, VAS, HHIA and psychoacoustic characteristics of tinnitus. Of those only the MML showed a significant inverse correlation with duration of tinnitus – that is, the longer the tinnitus the lower was the MML reduction. Greater shifts were seen in those with more recent onset of tinnitus. A possible explanation is that the longer the tinnitus duration, more central nervous system neural networks associated with it will be. In those cases, the MML measurement may show more resistance to change, pointing to greater neuroplasticity, hyperactivity and synchrony cross-talking occurring over the years. A longer period of stimulation and a larger sample size would be required to support our findings.

The direct and statistically significant correlation between THI, HHIA and MML scores reductions makes perfect sense, as it was previously also reported in several studies.^15^, ^31^, ^42^

The purpose of our study was to verify the effectiveness of HA to ameliorate tinnitus perception and related disturbance in a group of patients with chronic tinnitus and associated hearing loss, who had no prior experience with amplification. Those are a complex category of tinnitus patients. We believe that there is no other way to assess and verify tinnitus improvement, other than consider the impact of tinnitus on patient’s life quality, the difficulties caused by hearing loss, as well as the perception of tinnitus itself, as loudness. They are inseparable variables in the evaluation of any intervention for this subtype of patient.

## Conclusion

The evaluation parameters used in our study indicated tinnitus improvement in several aspects. THI, VAS and HHIA reduction scores estimated tinnitus and hearing loss impact in patient’s life. Loudness, Pitch and MML estimated tinnitus perception, and the decrease in MML values suggests decrease in tinnitus magnitude. We were able to show how these parameters are interrelated. Our study has provided evidence that HA fitting is a valuable treatment strategy for chronic tinnitus relief and associated hearing loss subtype of patient.

## Conflicts of interest

The authors declare no conflicts of interest.
